# Current Status of Poultry Recombinant Virus Vector Vaccine Development

**DOI:** 10.3390/vaccines12060630

**Published:** 2024-06-06

**Authors:** Haoran Wang, Jiaxin Tian, Jing Zhao, Ye Zhao, Huiming Yang, Guozhong Zhang

**Affiliations:** 1 National Key Laboratory of Veterinary Public Health Security, College of Veterinary Medicine, China Agricultural University, Beijing 100193, China; b20213050440@cau.edu.cn (H.W.); s20233051018@cau.edu.cn (J.T.); zhaoj@cau.edu.cn (J.Z.); yezhao@cau.edu.cn (Y.Z.); yanghm@cau.edu.cn (H.Y.); 2 Key Laboratory of Animal Epidemiology of the Ministry of Agriculture, College of Veterinary Medicine, China Agricultural University, Beijing 100193, China

**Keywords:** poultry, vaccines, fowl poxvirus, fowl adenovirus, Newcastle disease virus, Marek’s disease virus, herpesvirus of turkey

## Abstract

Inactivated and live attenuated vaccines are the mainstays of preventing viral poultry diseases. However, the development of recombinant DNA technology in recent years has enabled the generation of recombinant virus vector vaccines, which have the advantages of preventing multiple diseases simultaneously and simplifying the vaccination schedule. More importantly, some can induce a protective immune response in the presence of maternal antibodies and offer long-term immune protection. These advantages compensate for the shortcomings of traditional vaccines. This review describes the construction and characterization of primarily poultry vaccine vectors, including fowl poxvirus (FPV), fowl adenovirus (FAdV), Newcastle disease virus (NDV), Marek’s disease virus (MDV), and herpesvirus of turkey (HVT). In addition, the pathogens targeted and the immunoprotective effect of different poultry recombinant virus vector vaccines are also presented. Finally, this review discusses the challenges in developing vector vaccines and proposes strategies for improving immune efficacy.

## 1. Introduction

Currently, vaccination and biosecurity are the primary methods used to control the spread of poultry diseases. Among the various types of vaccines available, inactivated and attenuated vaccines have been widely utilized. With the advancement of recombinant technology and reverse genetic technology, recombinant virus vector vaccines have been developed, showing many unique advantages and gradually becoming the favorable choice compared with conventional vaccines (Table 1). Recombinant virus vector vaccines are created by incorporating one or more foreign genes, such as the viral protein 2 (VP2) gene of infectious bursal disease virus (IBDV), the hemagglutinin (HA) gene of avian influenza virus (AIV), the fusion (F) gene of Newcastle disease virus (NDV), or the fiber gene of fowl adenovirus (FAdV), into the nonessential region of viral replication using a live virus as a vector [1,2]. Because the foreign protein can be continuously expressed in the host, these vaccines can provide extended immune protection and induce specific cellular and humoral immune responses [3]. More importantly, some viruses, such as the herpesvirus of turkey (HVT), overcome the interference caused by maternal-derived antibodies [4,5,6]. Furthermore, such vaccines do not induce adverse reactions, show minimal risk of virulence reversion and satisfactory genetic stability, and have a low potential for horizontal transmission [2,7]. Moreover, these recombinant vaccines can be easily administered via subcutaneous injection at 1-day-old or in ovo [8,9]. Furthermore, the continual advancement of recombinant and reverse genetic technologies enables the rapid generation of recombinant viruses, exemplified by the creation of recombinant NDV within a remarkably short timeframe of weeks [10,11]. These advantages have contributed to the gradual adoption and application of recombinant virus vector vaccines. In addition, adenovirus, herpesvirus, and poxvirus have also been widely used. Since the start of the 21st century, numerous viral vector vaccines have been commercialized, such as Trovac^®^-NDV, Trovac^®^ AI H5, Vectormune^®^ FP LT, Newxxitek™ HVT + ND, VAXXITEK^®^ HVT + IBD + ILT, and Innovax^®^ ND-IBD.

This paper primarily describes the construction and characterization of poultry viral vector vaccines, the targeted pathogens, and the immunoprotective effects. Furthermore, we discuss the challenges in developing vector vaccines and propose strategies for immune efficacy.

## 2. Various Important Virus Vectors

### 2.1. Marek’s Disease Virus and Herpesvirus of Turkey

Marek’s disease (MD), caused by MDV, is characterized by highly contagious immunosuppression, the occurrence of malignant T-cell lymphomas, and the presence of neurological disorders in chickens [12]. Currently, three serotypes, namely, MDV serotype 1 (MDV-1), MDV serotype 2 (MDV-2), and MDV serotype 3 (MDV-3), also known as Mardivirus gallidalpha2, Mardivirus gallidalpha3, and Mardivirus meleagridalpha1, respectively, have been identified, which belong to the family Orthoherpesviridae, subfamily Alphaherpesvirinae, genus Mardivirus. MDV-1 encompasses oncogenic strains and their attenuated variants. The other two species, MDV-2 and MDV-3, are non-oncogenic viruses isolated from chickens and turkeys, respectively. The latter is also known as HVT [13]. Recently, the application of recombinant virus vector vaccines utilizing attenuated MDV-1 or HVT has garnered significant attention because of its ability to circumvent interference from maternal antibodies, induce persistent infection for lifelong immunity [12], and serve as an effective platform for developing multivalent vaccines that simplify vaccination schedules [2,14,15,16]. Moreover, the HVT-based vaccine has a unique advantage compared with other viral vectors: natural apathogenic, which makes it safer than attenuated MDV-1 [17] (Table 2).

**Figure 1:**
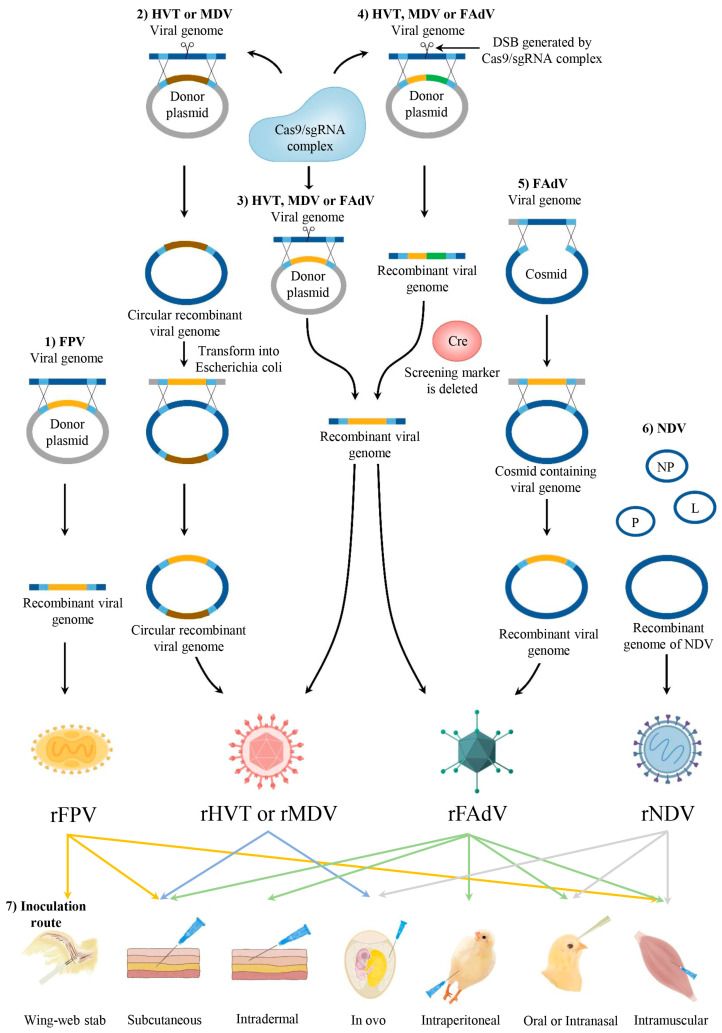
Schematic representation of the commonly used methodologies (note: this is not an exhaustive list) to obtain recombinant viral vectors and the inoculation routes of different recombinant viruses. (1) Construction of recombinant FPV by homologous recombination. The foreign gene (yellow box) is contained in a donor plasmid, which harbors sequences (light blue box) complementary to the target site within the vector genome, completing the insertion of the foreign gene through homologous recombination. (2) Construction of recombinant HVT or MDV using the BAC system. Recombinants can be obtained by inserting BAC vector sequences (brown box) into the nonessential replication region by homologous recombination, followed by extracting the circular DNA at the stage of rolling-circle replication, then transforming the viral genome into Escherichia coli. The viral genome can be manipulated by homologous recombination. (3) Construction of recombinant HVT, MDV, or FAdV by the CRISPR/Cas9 system. A double-strand break (DSB) is generated by cleaving the target sequence using the Cas9/sgRNA complex, followed by the insertion of a screening marker contained in the donor plasmid into the viral genome through homologous recombination. The screening marker is then replaced with the foreign gene in the same way. (4) Construction of recombinant HVT, MDV, or FAdV by the Cre-LoxP system. A DSB is generated by cleaving the target sequence using the Cas9/sgRNA complex, followed by the insertion of foreign genes and a screening marker (green box) contained in the donor plasmid into the viral genome through homologous recombination. Then, the screening maker can be deleted by Cre recombinase. (5) Construction of recombinant FAdV using the cosmid system. The FAdV genome and a cosmid vector carrying the left and right homology arms were co-transformed into *E. coli* to generate infectious clones by homologous recombination. Subsequently, the recombinant virus was generated by linearizing the infectious clone with restriction enzymes and transfecting the cells. The replacement of the foreign gene can be performed based on infectious clones using a screening marker. (6) Construction of recombinant NDV. Recombinant full-length antigenomic cDNA from NDV and plasmids encoding the NP, P, and L viral proteins are transfected into BHK-21 cells. (7) Common inoculation routes of the different recombinant viruses mentioned in this article. Recombinant FPV can be inoculated by wing-web stab or via the subcutaneous or intramuscular routes; recombinant HVT or MDV can be inoculated via the subcutaneous or in ovo routes; recombinant FAdV can be inoculated via the subcutaneous, intradermal, intraperitoneal, or oral routes; recombinant NDV can be inoculated via the intramuscular, oculo-nasal, or in ovo routes.

The genomes of MDV-1, MDV-2, and HVT, which are double-stranded DNA of approximately 178 kbp, consist of a unique long (UL) segment and a unique short (US) segment, which are flanked by terminal and internal repeats long (TRL and IRL) and terminal and internal repeats short (TRS and IRS), respectively [18]. About six frequently used nonessential regions for viral replication in the genome provide insertion sites for foreign expression cassettes, including the US2 and US10 genes, the UL45-UL46, HVT005-HVT006, HVT065-HVT066, and SORF3-US2 intergenic regions of the HVT genome [15,19,20,21], and the US2 and UL41 genes of the MDV-1 genome [22]. The strategy for generating recombinant MDV-1 and HVT may involve the utilization of bacterial artificial chromosomes (BACs) and homology-directed repair (HDR)-dependent CRISPR/Cas9 or fosmids. Each strategy has unique advantages based on its characteristics. In the CRISPR/Cas9 system, one plasmid carrying the expression cassette flanked by upstream and downstream homology arms was required to co-transfect with a commercially available plasmid carrying sgRNA and Cas9 gene into cells through HDR-dependent CRISPR/Cas9 strategy and followed by infecting HVT or MDV. Upon appearing with a cytopathic effect, the recombinant virus can be obtained through plaque purification [19,23]. In recent years, the Cre-loxP system combined with Non-homologous DNA end joining (NHEJ)-dependent CRISPR/Cas9 has also been applied to rescue recombinant viruses [21,24,25,26]. This approach is utilized due to the higher efficiency of NHEJ in introducing foreign genes, as NHEJ can occur throughout the cell cycle [27]. In contrast, HDR is limited to the S and G2 phases [28,29,30]. In addition, the introduction of Cre-LoxP technology has made it more convenient to excise reporter-gene precisely [25]. Neither method CRISPR/Cas9 nor the Cre-LoxP system requires the construction of the reverse genetic system of HVT, which seems convenient, but the process of plaque purification is time-consuming, probably taking weeks or even months. Furthermore, the genome may be mutated as HVT passes through the generations. Therefore, many scholars have tried to build the reverse genetic system of HVT, although it is very difficult to generate. For the BAC system, recombinants can be obtained by inserting BAC vector sequences into the nonessential replication region by homologous recombination, followed by extracting the circular DNA at the stage of rolling-circle replication [31] (Figure 1). A significant advantage of the BAC strategy is that the viral genome can be easily manipulated in bacteria by transforming the recombinant into *E. coli*. Different recombinant viruses can be produced in high throughput and only in a matter of weeks. However, it is important to note that BAC vector sequences in the viral genomes may lead to characteristic alterations in the recombinant viruses [32,33]. It can optionally be knocked out by the CRISPR/Cas9 system [34] or can be overcome by using the fosmid strategy, which involves excision using restriction before the transfection [35,36]. However, the BAC and fosmid strategies must avoid the laborious steps of constructing plasmids that carry viral genomes. In recent months, a simple, rapid, and efficient method based on single-step transformation-associated recombination (STAR) in Saccharomyces cerevisiae was used to generate complete betaherpesvirus and gammaherpesvirus genomes [37]. Conveniently, the linear viral genome can be directly integrated into a vector comprising a yeast centromeric plasmid and a BAC replicon, which avoids the laborious steps of generating plasmids carrying viral genomes. Currently, no relevant reports exist that this method is applied to the reverse genetics system of HVT or MDV. However, this method undoubtedly provides a new direction in constructing reverse genetic systems of large genome DNA viruses. Theoretically, cracking infected cells and obtaining virions by ultracentrifugation, then extracting complete viral DNA, which could finally link with BAC plasmids through the STAR system, may quickly obtain clones of the complete HVT or MDV genome.

Assessing the effectiveness of vaccines has consistently been based on the level of immune protection. Numerous studies have demonstrated that the immune protection offered by vaccines is primarily influenced by the insertion site and promoter, both of which affect the expression level of foreign proteins. Gao et al. demonstrated that insertion of the expression cassette carrying the HA gene into the US2 and US10 genes resulted in recombinant viruses exhibiting distinct biological properties, such as plaque morphology and size, growth kinetics (the growth of rHVT-US10-HA was higher than rHVT-US2-HA within 54 h, but lower than rHVT-US2-HA after 54 h), and protective efficacy (the antibody titer induced by rHVT-US10-HA was lower than that induced by rHVT-US2-HA, which might have induced better immune protection) [20]. Similarly, a study by Zai et al. confirmed that different insertion sites could affect the expression of foreign proteins, thereby affecting the immune protection effect [38]. In MDV, the work of Zhang et al. revealed that different sites in recombinant MDV showed different transcriptional activities for the expression cassette of the H9N2 AIV HA gene [39]. Differences in transcriptional activities can result in varying protein expression levels, thereby influencing the immunoprotective effect. CMV, Pec (CMV/β-actin chimera promoter), EF1α, CAG, and SV40 are commonly used promoters in foreign expression cassettes. Tsukamoto et al. constructed two HVT recombinants expressing the VP2 protein of IBDV using the CMV and Pec promoters, respectively, and found that rHVT-pecVP2 expressed the VP2 antigen at approximately four times higher levels than rHVT-cmvVP2 in vitro. Furthermore, there was a positive correlation between the protein expression levels, the antibody levels, and the immunoprotective effect [40]. Similarly, Li et al. revealed that the protein expression, antibody level, and immune protection of the MDV recombinant expressing the VP2 protein of IBDV under the Pec promoter was higher than that of the equivalent recombinant under the CMV promoter [35]. These results support the conclusion that higher protein expression levels lead to enhanced immune protection. However, according to the experimental findings of Sonoda et al., although the protein expression level of recombinant MDV-1 expressing the F protein of NDV under the control of the simian virus 40 late promoter [rMDV1-US10L(F)] was significantly higher than [rMDV1-US10P(F)] utilizing the MDV1 glycoprotein B (gB) promoter, rMDV1-US10P(F) induced faster and stronger antibody responses to NDV-F and MDV1 antigens in commercially available chickens compared with rMDV1-US10L(F). Furthermore, administering rMDV1-US10P(F) to commercial chickens with existing maternal antibodies resulted in complete protection against NDV challenge [41,42]. Moreover, the transcriptional activity and protective efficacy of five distinct promoters were assessed in recombinant MDV, encompassing three MDV endogenous promoters [specifically the promoter for the gB gene, a bi-directional promoter for pp38 (ppp38), and a promoter for 1.8 kb RNA transcripts (p1.8 kb)], as well as the CMV and SV40 promoters, all controlling the HA gene of H9N2. The findings revealed that the gB gene promoter and ppp38 demonstrated notably lower transcriptional activities, particularly the ppp38 promoter, which conferred 50% protection against challenge, while the gB promoter provided 25% protection. Conversely, the remaining three promoters exhibited relatively high transcriptional activity and did not elicit any immunoprotective effect [43]. In summary, there is no direct correlation between the expression of certain antigens and the immune effect, which may be attributed to excessive protein expression surpassing the optimal concentration for antibody production, leading to decreased efficiency. Alternatively, a high level of expression may trigger an excessive immune response, inhibiting the growth of the recombinant virus in the host and ultimately compromising immune protection. These findings suggest that screening promoters are crucial for achieving improved immunoprotection.

Because of their well-established safety profile and its convenient route of immunization (in ovo), the HVT-based vaccines have gained significant popularity for expressing protective antigens of different avian pathogens. At present, compared with other virus vector vaccines, HVT has the best commercial viability. To date, over a dozen HVT-based vaccines have become commercially available, including VAXXITEK^®^ HVT + IBD, Newxxitek™ HVT + ND, Vectormune^®^ HVT ND, Vectormune^®^ AI H5, Vectormune^®^ AI H7, Innovax^®^ ND, and Procerta^®^ HVT-ND. However, it is crucial to emphasize that administering two different rHVT vaccines to the same bird is not recommended as the immune response generated by the virus with faster replication can neutralize the virus with slower replication, which can significantly compromise the effectiveness of both vaccines. Therefore, commercially available rHVT vaccines expressing multiple antigens have been developed, including Innovax^®^ ND-ILT, VAXXITEK^®^ HVT + IBD + ILT, VAXXITEK^®^ HVT + IBD + ND, Ultifend^®^ IBD ND, and Innovax^®^ ND-IBD. These double-insert live vaccines have been optimized to provide effective immune protection against a variety of pathogens. For example, Innovax^®^ ND-IBD can provide 8 weeks of immunity duration against ND and IBD and an entire risk period of immunity duration against MD. Ultifend^®^ IBD ND can provide 9 weeks of immunity duration against ND (18 weeks for layer chickens) and IBD for broiler chickens.

In addition to commercially available vaccines, numerous recombinant HVTs or MDVs carrying different foreign proteins, such as the VP2 protein of IBDV, influenza HA, the PmpD protein of C. psittaci, the F protein of NDV, and the gD and gI proteins of ILTV, have been assessed for their immunoprotective effects in the last decade (Table 3). There is a greater emphasis on developing recombinant vaccines using HVT as a vector than MDV, which may be attributed to the safety profile of rHVT. However, since the continued evolution of MDV toward greater virulence, it has become less effective in preventing very virulent (vv) and very virulent plus (vv+) MDV strains [17]. This drawback can be overcome by co-immunizing with HVT and MDV-2 [44] or using MDV-1 vaccine [45]. Furthermore, combined immunization with rHVT and rMDV, expressing different foreign genes, has been demonstrated to offer effective immune protection. Studies have shown that a combination of an HVT vaccine expressing NDV antigen and an MDV serotype 1 Rispens virus expressing IBDV antigen offered 94%, 100%, or 94% protection against MDV, IBDV, or NDV challenge, respectively, without any notable interference [46]. Alternatively, the administration of HVT with a plasmid containing chIFN-γ or chIL-17A has been shown to enhance the protective effect against vv MDV strains [47,48]. Indeed, current MD vaccines do not elicit sterilizing immunity or effectively inhibit the harboring of challenge viruses [49]. Vaccines that allow the host to survive but do not prevent pathogen spread would allow the vv strains to emerge and persist. This type of vaccine is often called a leaky vaccine [50]. Gandon et al. demonstrated that vaccines that partially suppress viral growth may cause exceptionally high levels of evolutionary change in the wild-type pathogen [51]. This type of vaccine may offer only a temporary solution. Therefore, developing vaccines against MD that can effectively block shedding should be a primary focus.

### 2.2. Fowl Poxvirus

The capacity for large exogenous genes renders poxvirus-based vector systems widely applicable platforms for use in vaccine development. These systems have been extensively leveraged to formulate recombinant vaccines encoding various antigens to mitigate the onset of diseases in both human and animal populations [52], especially vaccinia virus, which has been evaluated for efficacy against various viruses, for example, dengue virus [53], Zika virus [54], ebolavirus [55], and influenza A virus [56,57]. The basic vaccinia virus vector technology has been extended to other members of the poxvirus family, particularly fowl poxvirus (FPV), which was targeted for species-specific applications in the poultry industry. According to the latest classification results published by the International Committee on Taxonomy of Viruses (ICTV), FPV, along with canarypox virus, pigeonpox virus, psittacinepox virus, and others belonging to the genus Avipoxvirus, of the subfamily Chordopoxvirinae are large, enveloped, brick-shaped DNA viruses with genomes of approximately 300 kb [58].

Avipoxvirus-based vector systems have several remarkable characteristics. First, because of the poxviral high-fidelity DNA polymerase, they are genetically stable with low nucleotide mutation rates compared with other viruses [59]. Second, the large genome containing several nonessential regions for viral replication allows for the integration of multiple foreign genes [60], which enables the development of multivalent vaccines. However, it is important to note that replication of the virus and translation of proteins is accomplished by enzymes encoded by the poxvirus, independent of host-cell enzymes. Thus, the transcription of foreign genes must employ poxvirus promoters. Third, poxvirus implements its replication process within the confines of the cytosol, thereby eluding the inherent risk of recombination with the genes of its host organism [61]. Fourth, the infection induced by poxvirus in mammalian hosts exhibits a transient nature, precluding the possibility of being transmitted to other animals or disseminating into the surrounding environment [62] (Table 2).

The methodologies employed in genetic engineering for the fabrication of recombinant FPV predominantly use the homologous recombination process to incorporate exogenous genes into the vector genomes. To elaborate, the expression cassettes of these foreign genes are conveyed via a transfer plasmid. This plasmid harbors sequences complementary to the target site within the vector genome, completing the insertion of the expression cassette through homologous recombination [63,64] (Figure 1). Moreover, the CRISPR/Cas9 system has been used to engineer the orthopoxvirus [65] and canarypox virus [66]. However, this system has yet to be applied to FPV.

To our knowledge, the first recombinant FPV used to prevent poultry viral diseases was developed by inserting the HA gene of an H5N8 strain into the FPV strain FP-1 genome in the 1980s, which provided complete protection against homologous (H5N8) and heterologous (H5N2) influenza viruses [67]. Following this significant achievement, several recombinant poxvirus vaccines obtained licensure in different countries, including Trovac^®^-NDV, Trovac^®^ AI H5, Trovac^®^ Prime H7 (Boehringer Ingelheim Animal Health), Vectormune^®^ FP ND, and Vectormune^®^ FP-LT (Ceva Animal Health). Trovac^®^-NDV, which can induce high levels of antibodies maintained for 8 weeks in specific-pathogen-free (SPF) chickens through a single dose, can protect against both a lethal intramuscular NDV challenge and an FPV challenge in the presence of maternal antibodies. This vaccine has received authorization for administration via subcutaneous routes at one day of age. Despite this, its adoption in field applications remains limited [68]. Furthermore, to protect against the H5 subtype influenza virus, an additional vaccine from Boehringer, referred to as “Trovac^®^ AI H5”, was accorded emergency authorization in the USA in 1998. Subsequently, it has achieved full registration in numerous countries. The Trovac^®^ AI H5 vaccine protected against mortality (90–100%), morbidity (90–100%), and cloacal and respiratory shedding of highly pathogenic avian influenza (HPAI) Mexican H5N2 isolate [69,70]. In addition, the efficacy of Trovac^®^ AI H5 has been assessed against various HPAI isolates. It conferred comprehensive protection against clinical symptoms and mortality following exposure to nine distinct HPAI H5 viruses. These viruses, collected over 38 years, exhibited 87.3–100% sequence similarity with the HA protein in the vaccine [71]. The presence of maternal immunity against fowl pox and maternal antibodies to AIV did not notably impede the induction of immunity in progeny vaccinated with the Trovac^®^ AI H5 vaccine at one day of age [72]. The protection conferred against the HPAI virus challenge demonstrated inconsistency when administered as a secondary immunization following a primary control FPV vaccine FP-C vaccination. Pre-existing immunity could inhibit the live vaccine from initiating an infection, subsequently obstructing the successful establishment of an immune response; however, both vaccines protected against a virulent fowl pox challenge [73]. Therefore, the vaccine is not recommended in birds previously vaccinated or infected with fowl pox. This is one of the limitations of the use of recombinant FPV (rFPV) in poultry. Another limitation is that vaccine and field strains of FPV can be integrated with remnants of the reticuloendotheliosis virus (REV) long terminal repeats or the near full-length genome of REV [74]. Previous studies have shown that FPV vaccine contamination with REV caused tumors in the vaccinated birds [75,76,77], and the nearly intact integrated REV provirus could likely engender infectious REV, which, consequently, may lead to immunosuppression, thereby escalating the pathogenicity [77,78]. However, the mechanisms involved have not been fully elucidated [79,80]. Contamination of FPV vaccine with avian leukosis virus (ALV) is also considered a serious problem. A retrospective survey of different batches of attenuated vaccines produced before 2010 in China showed that three ALV strains that shared the highest homology (97.7%) with the wild ALV-A strains isolated in China during the same period were successfully isolated from three attenuated vaccines, respectively, including a live FPV vaccine, a live NDV vaccine, and a live IBDV vaccine [81]. Similarly, the nucleic acid of ALV-J was detected by PCR in commercially available FPV vaccines in Nigeria [82]. This phenomenon suggests that ALV can infect chickens via vaccination with live vaccines, which requires us to adopt strict testing measures to detect exogenous viruses after all vaccine production.

In addition to using commercial vaccines from different companies, several candidate strains have been evaluated for protective efficacy (Table 3). Chen et al. selected the gB gene from predominantly epidemic strains and inserted it into the FPV (an FPV vaccine strain 282E4) genome to construct the recombinant virus rFPV-gB. The protective index of rFPV-gB in SPF chickens was 100% for mortality, surpassing that of the crFPV (commercial infectious laryngotracheitis virus (ILTV) recombinant FPV engineered vaccine) (83.3%) in SPF chickens. The genetic distance between the challenge strain and the commercial vaccine strain may cause this difference in protection. However, neither commercial vaccine nor rFPV-gB was effective in reducing virus shedding, and vaccines that fail to prevent shedding may create selective pressure, leading to the emergence of more virulent ILTV strains, ultimately resulting in the lower protective efficacy of existing vaccines [83]. Information on other recombinant FPVs as immunogens is presented in Table 3.

Studies have demonstrated that utilizing FPV vectors encoding cytokines, including interleukin (IL)-6 or gamma interferon, is a reliable and efficient vaccine strategy that can be employed to selectively modulate the immune response [84,85,86]. Alternatively, IL-18 has the potential to enhance the protective effect. Chen et al. inserted chicken IL-18 into the nonessential open reading frame (ORF) 030 of a live recombinant FPV vaccine (fpIBD1) against IBDV while knocking out potential IL-18 binding proteins (ORF FPV073 and ORF FPV214). The results showed that compared with the original fpIBD1, which could not avoid the bursa damage caused by IBDV infection, both fpIBDIΔ073::IL-18 and fpIBD1Δ214::IL-18 provided complete protection after IBDV challenge, with no bursal damage or IBDV detected in the bursae of the birds [63] (Table 3). In summary, co-expression of cytokines and other immunomodulatory factors can improve the immune protection of recombinant FPV. However, it is important to remain vigilant that chickens and wild birds that have been vaccinated with live attenuated FPV vaccine still have the potential for an Avipoxvirus outbreak, which may be related to climate conditions, breeding density, broad host range, or immunosuppressive virus co-infection. These findings indicate that permanent vigilance and improved sanitary and vaccination programs are necessary [87,88].

### 2.3. Fowl Adenovirus

According to the latest ICTV classification, FAdV belongs to the family Adenoviridae and genus Aviadenovirus. Virus particles of the family Adenoviridae have icosahedral morphology and are nonenveloped; the genome is a 26–45 kbp linear double-stranded DNA molecule [89]. Because adenoviruses can effectively introduce large foreign DNA molecules into target cells, allow for the packaging of formulations as sufficiently stable lyophilized preparations, and have robust protocols available for the construction and production of high-titer virus in vitro, some adenoviruses have been developed as recombinant vaccines to prevent poultry diseases [90] (Table 2). Based on the sera cross-neutralization assay or their molecular structure, FAdV is further divided into 12 serotypes (FAdV-1–8a, 8b–11) or five species (FAdV-A–E), respectively [91,92]. In poultry farming, FadV-4 is associated with hepatitis-hydropericardium syndrome (HHS), which leads to high mortality in chickens and causes significant economic losses, showing widespread prevalence according to epidemiological investigations [93]. Especially over the last decade, there has been an increasing trend toward endemic outbreaks in many countries rather than sporadic occurrences [94,95]. Therefore, FAdV-4 is widely utilized as a platform for recombinant vaccine development [96,97]. In addition, FAdV-8 [98], FAdV-9 [99], FAdV-10 [100], and HAdV-5 [101] have also been developed as recombinant vaccines for preventing poultry disease.

Developing recombinant DNA technology has enabled more efficient strategies for recombinant FAdV generation, such as cosmid [102], fosmid [103], CRISPR-Cas9 [104,105], and Cre-LoxP [106] systems, which are common strategies for constructing recombinant viruses. For example, in cosmid-based strategies, the adenovirus genome and a cosmid vector carrying the left and right homology arms were co-transformed into Escherichia coli to generate infectious clones by homologous recombination. Subsequently, the recombinant virus was generated by linearizing the infectious clone with restriction enzymes and transfecting the cells. The replacement of the foreign gene can be performed based on infectious clones using a screening marker [102] (Figure 1). Similarly, recombinant viruses can also be constructed utilizing the fosmid-based system [103].

Recent studies have shown that the N and C termini of fiber-2 and the N-terminus of fiber-1 of FAdV-4 could be potential insertions for developing live-attenuated vaccine candidates against FAdV-4 or vaccine vectors for expressing foreign antigens [107,108]. For example, Guo et al. successfully engineered a recombinant virus FAdV4-HA(H9), which expresses the HA gene of the H9N2 influenza virus by replacing the shaft and knob region of fiber-2. Subsequent animal studies demonstrated that this recombinant virus exhibited attenuation and elicited a hemagglutination inhibition response against the H9N2 influenza virus at an early stage, effectively suppressing viral replication in the oropharynx. Nevertheless, this recombinant virus can cause a 25% mortality rate [106] (Table 3). Furthermore, fiber-2 has recently been confirmed to not be necessary for either virus replication or efficient protection against FAdV-4, which means that fiber-2 can be used as a target site to be replaced with different foreign genes for generating an FAdV-4-based vaccine vector against other pathogens [105,109]. Thus, through replacing the fiber-2 gene of FAdV-4 with the fiber gene of FAdV-8a, a novel recombinant virus FAdV4-F/8a-rF2 was generated, which could induce high levels of neutralizing antibodies and provide 100% protection against mortality for both FAdV-4 and FAdV-8a. Moreover, the recombinant virus was nonpathogenic to SPF chickens [97] (Table 3). Similarly, Guo et al. obtained a novel recombinant FAdV-4 expressing the fiber-2 protein of duck adenovirus 3 (DAdV-3) by the same strategy. The author evaluated some characteristics of this recombinant and found that inoculation of a high dose of recombinant virus did not cause any clinical symptoms in the infected two-week-old SPF chickens. Moreover, a high antibody level against FAdV-4 and DAdV-3 with neutralizing titers could be induced. However, the protective effect against FAdV-4 and DAdV-3 was not evaluated [110]. Recombinant virus vector vaccines were always expected to have a high expression level of exogenous protein to mount an effective immune response. However, research has shown that there may be no association between foreign protein expression levels in vitro and the induction of antibody production levels. James et al. engineered different recombinant FAdV-9 with enhanced green fluorescent protein (EGFP) gene expression cassettes carrying the CMV, CAG, or EF1α promoters. After infecting hepatoma cells, the recombinants carrying the CAG or EF1α promoter had a higher protein expression level than the recombinant carrying the CMV promoter, which, surprisingly, had the highest seroconversion to EGFP in chickens. This may be because the high level of foreign protein expression under the CAG promoter may have exceeded the highest threshold dose for antibody production [111]. These findings suggest that candidate recombinant virus vector vaccine selection should be based on the level of humoral immune response rather than the level of foreign protein expression in vitro [112].

FAdV-8, which is associated with inclusion body hepatitis (IBH), was also developed as a virus vector. Johnson et al. generated two FAdV-8 recombinants by inserting the S1 gene of infectious bronchitis virus (IBV) between the SnaBI and XbaI restriction enzyme sites or between the SpeI sites of the CFA40 strain. Animal experiments demonstrated that oral vaccination at day 6 could induce 90–100% protection against either homologous or heterologous challenge at the trachea [98] (Table 3).

Other serotypes of FAdV have also been used as vaccine vectors to express exogenous proteins. For the prevention of infectious bursal disease (IBD), the VP2 gene expression cassette containing the CMV promoter was inserted at the left end of the CELO (FAdV-1) genome to generate CELOa-VP2 vector, which could provide complete protection against mortality and morbidity associated with highly virulent IBDV when injected once or twice subcutaneously or intradermically but not oro-nasally [113] (Table 3). Similarly, FAdV-10 was used as a virus vector to express the VP2 protein, and the recombinant could protect chickens against challenge with an IBDV strain of intermediate virulence through intravenous, intraperitoneal, subcutaneous, or intramuscular injection but provided no protection when delivered via the conjunctival sac [100] (Table 3). Information on other recombinant FAdVs as immunogens is presented in Table 3. Although many FAdV vaccine candidates have been reported, a commercially accessible recombinant FAdV vaccine remains unavailable.

In addition to FAdV, a series of research studies show that replication-deficient human adenovirus serotype-5 (HAdV-5) is more suitable to deliver foreign proteins as a vaccine vector because of the following significant advantages: (1) Because HAdV-5 has shown no immunity in chickens, it can effectively avoid the interference of maternal antibodies [114]; (2) The HAdV-5 vector does not result in the spread of the virus because it cannot replicate in animals [115]; (3) As the HAdV-5 genome does not integrate into the host genome, the HAdV-5 vector cannot interfere with the expression of other host genes [115,116]; (4) Finally, the HAdV-5 vector can be administered via different routes [117]. Xu et al. constructed three HAdV-5 recombinants using the AdEasy system, which has been developed as a kit and is commercially available: (1) rAd5-F: expressing only the NDV (genotype VII NDV strain DHN3) F protein; (2) rAd5-VP2: expressing only the IBDV VP2 protein; and (3) rAd5-VP2-F2A-F: co-expressing the F and VP2 proteins. All rAd5 constructs were used in immunization via intramuscular injection. Animal experiments revealed that rAd5-F and rAd5-VP2-F2A-F provided 100% and 86% survival rates after challenge with NDV strain DHN3, respectively, for SPF chickens. However, neither recombinant prevented viral shedding. Both rAd5-VP2 and rAd5-VP2-F2A-F provided an 86% survival rate after challenge with IBDV strain BC85/86. All recombinants could induce efficient cellular immune responses in SPF chickens similar to the attenuated strain rDHN3-mF and the commercial HVT-VP2 vector vaccine but had weaker induction of antibody responses, which might be due to the antibody detection method. However, a more plausible explanation is that the replication defect in HAdV-5 results in the low expression of foreign protein in chickens to stimulate a weaker humoral immune response, which could be improved by increasing the dose and immunization times of the recombinant adenoviruses [101]. Given the potential of HAdV-5 as a platform for recombinant vector development, multiple reports have specifically investigated recombinant HAdV-5-expressing antigens from viruses, including NDV [101], Mycoplasma gallisepticum [118], IBDV [101], and Chlamydophilia psittaci [119]. A drawback of HAdV-5 recombinants is their potential for recombination with the E1 region of the human 293-derived cell genome, resulting in recombinant viruses with replication capability. However, similar recombination was not found in PER.C6 or CER8 cells, which are suitable for producing HAdV-5 recombinants [120].

### 2.4. Newcastle Disease Virus

Newcastle disease (ND), which is caused by NDV, is a highly contagious viral disease associated with significant economic losses in the poultry industry [121]. According to the latest ICTV classification, NDV, also known as Orthoavulavirus javaense, is a member of the genus Orthoavulavirus in the subfamily Avulavirinae. Despite the increased implementation of vaccination strategies in numerous countries, outbreaks of ND still occur frequently [122,123,124]. Therefore, the prevention of NDV should continue to be a primary focus in the poultry industry. In recent decades, LaSota and other NDV vaccine strains, including I-2, B1, Clone 30, and VG-GA, have been extensively applied and have made significant strides against ND. Furthermore, some have been employed as vaccine vectors to effectively express heterologous proteins [125,126,127] because of the following significant advantages: (1) NDV has a relatively short and simple genome of approximately 15 kb that encodes six structural proteins; (2) The reverse genetics system of NDV has been extensively used to generate attenuating NDV vector candidates; (3) NDV demonstrates efficient replication in the respiratory tract of primates, inducing both mucosal and systemic immune responses; (4) Because NDV replicates in the cytoplasm, the NDV genome does not integrate into the host genome. Moreover, recombination events involving the NDV genome are exceedingly rare; (5) The genome of NDV can express multiple foreign genes, and the expression level can be modulated by manipulating the insertion site (Table 2). The expression of a foreign gene was found to be higher when the insertion site was closer to the 3′ end (except between the NP and P genes), but the growth kinetics were slightly delayed [128,129,130]. The first reverse genetic system for NDV was established by Peeters et al. in 1999. Briefly, three plasmids expressing the NP, P, and L proteins, respectively, and a plasmid containing the NDV complete genome, which was under the control of the T7 promoter, were co-transfected into cells that were infected with a recombinant FPV that expressed T7 RNA polymerase (FPV-T7) [131], leading to the synthesis of the antigenome RNA of NDV. The NP, P, and L proteins were responsible for the replication and transcription of the antigenome RNA. Finally, SPF chicken embryos propagated the recombinant virus [132]. In recent decades, the NDV reverse genetics system has been developed and continuously improved, primarily focusing on the acquisition of T7 RNA polymerase and optimization of the promoter for the plasmid containing the NDV complete genome. For example, a highly attenuated host-range-restricted modified vaccinia virus Ankara [133], which is incapable of replicating in humans and most other mammalian cells but supports replication in avian cells such as CEF cells, was used for expressing T7 polymerase [134], which resulted in a lower cytopathic effect and enabled sufficient time for T7 polymerase expression. More importantly, biosafety concerns relating to the vaccinia virus were reduced [135,136]. In addition, BSR T7/5 cells that can express the phage T7 RNA polymerase stably in culture have been used to simplify the NDV reverse genetics system [137]. In addition to the T7 promoter, promoters that can be recognized by RNA polymerase II, such as CMV, have also been used to transcribe the antigenome RNA of NDV. Meanwhile, the hammerhead ribozyme sequence and hepatitis delta virus ribozyme sequence were inserted into the 5′ and 3′ ends of the antigenomic sequence, respectively, to obtain antigenomic RNA with exact termini. Studies have shown that the CMV promoter system based on BHK-21 cells facilitates the generation of rNDV and exhibits greater efficiency compared with the T7 rescue system based on BSR-T7 cells [138]. The reverse genetics system for NDV has become remarkably simple and efficient as a result of these optimizing strategies. However, it still presents certain challenges. Most noteworthy, the complexity and length of the genome have rendered the generation of full-length high-fidelity cDNA the most arduous and time-consuming step during the rescue of NDV. To overcome this challenge, ligation-independent cloning was used to construct NDV full-length cDNA clones [10,139]. Yu et al. designed two pairs of NDV “genotype-universal” primers according to the conserved genomic regions of different genotypes of NDV strains to generate three full-length cDNA clones, including LaSota, VG/GA, and F48E9, using the two-step ligation-independent cloning strategy. Compared with conventional cloning approaches, this strategy significantly reduced the number of cloning steps and shortened the time required to rescue NDV to weeks [10]. The low probability of the successful transfection of four different plasmids into one cell and the generation of at least one viable and replicable viral particle are other factors that contribute to the challenge of rescuing NDVs by conventional reverse genetics. Liu et al. reported an improved rescue strategy that only requires two plasmids, pNPL and an NDV full-length clone, under the control of the CMV promoter. This system enabled the rescue of NDV through the co-transfection of the two plasmids into BHK-21 cells, followed by the collection of the cells and supernatant for virus amplification in eggs. Two significant advantages of this strategy are time-saving and the ability to rescue viruses that are unable to take up four plasmids, particularly for attenuated strains [11] (Figure 1). Recently, a novel system has been developed to rescue a virus from a single plasmid. This system strategically inserted a T7 promoter sequence before the P and L genes. Following transfection of the plasmid into cells infected with a fowl pox virus expressing T7 RNA polymerase, three distinct RNA species were generated, including a full-length viral RNA and two subgenomic RNAs. Subsequently, these mRNA transcripts were utilized to synthesize NP, P, and L proteins, producing infectious virus particles. Since only one plasmid was required, this system greatly improved the rescue efficiency of the virus and has important application value in the reverse genetics of NDV [140].

The evaluation of rNDV as an experimental vaccine for poultry has been conducted over the past 20 years. Various rNDVs expressing influenza HA glycoproteins [96,141,142,143], FAdV-4 fiber-2 protein [96], IBDV VP2 protein [125,126], IBV S protein [144,145,146], MDV gB protein [147], ILTV gD protein [148], and σC protein [149] have been generated and evaluated for their immunoprotective effects in chickens. Information on rNDVs used as immunogens is presented in Table 3. Some research has suggested that LaSota remained the favored option for developing recombinant viral vectors based on its ability to induce strong immunoprotection and its safety profile.

Furthermore, research has been conducted to optimize the genome of LaSota. For example, substituting the HN gene with that of a thermostable strain results in the acquisition of thermostability, thereby reducing vaccination costs, which especially benefits developing and less-developed nations [144]. This is a unique advantage of NDV as a vaccine vector. In addition, better protection could be achieved by replacing the F or HN with the corresponding genes of local epidemic strains [150]. The intergenic regions between the P and M genes are considered popular insertion sites for foreign genes because they not only ensure efficient expression of the foreign protein but also have little impact on the replication efficiency of the recombinant virus compared with the parental virus [128,129]. Despite providing effective protection, there are few commercially available vaccines except for a recombinant ND vaccine containing an H5 AI hemagglutinin gene insert (Harbin Weike Biological Technology Development, Harbin, China). One of the most important reasons is the interference from maternally derived antibodies, which restrain the utilization of NDV vector vaccines in poultry. Many efforts have been taken to overcome MDA interference, including higher antigen doses, more aggressive vaccine strains, and restoration of B cell response by cytokine stimulation [151]. In the future, more progress and substantiation of the contribution of the recombinant NDV is expected.

### 2.5. Other Recombinant Virus Vectors

Besides the above-mentioned commonly used virus vectors, IBV, ILTV, and Duck enteritis virus (DEV) are also increasingly being used as viral vectors to control common viral diseases in poultry.

According to the latest version of ICTV, IBV belongs to the subgenus Igacovirus, genus Gammacoronavirus, subfamily Orthocoronavirinae, and family Coronaviridae, which has a linear and positive single-stranded RNA genome approximately 27–28 kb long [152]. The large size of IBV genomes, their capability for foreign gene expression, and the continual requirement for IBV vaccination make them suitable for use in developing viral vector vaccines [153]. Bentley et al. successfully replaced three different IBV genome regions, Gene 5, ORFs 3a, and 3b or the IR, with reporter genes eGFP or hRluc, and all recombinant viruses could successfully express reporter genes. Notably, the recombinant virus with Gene 5 replaced exhibited the greatest stability, presenting that IBV has potential as a vaccine vector for other avian pathogens [153]. Subsequently, Yang et al. replaced the 5a gene of IBV-H120 with the HN gene of NDV-LaSota and generated a recombinant IBV virus that provided 80% protection against challenges with virulent IBV and NDV. The level of protection achieved was comparable to that of commercial vaccines [154]. Furthermore, recent studies have shown that replacing S1 genes with the corresponding genes of different strains could improve the cross-protection efficiency of the IBV recombinant [155,156]. For example, replacing the partial S1 gene of the Beau strain with that of the QX-like strain can provide broad-spectrum protection against two different genotypes of IBV, which also proves its potential as a viral vector [156].

Earlier studies have shown that the infectious laryngotracheitis virus with large genomes also has the potential as a viral vector, which has been used to express the H5 or H7 protein of the influenza virus and obtain effective immune protection [157,158,159]. Furthermore, ILTV live vaccines are easily administered to large populations of animals through convenient methods such as eye drops, aerosol, and drinking water. However, recombinant ILTV vaccines are not widely available currently, possibly because of the lower efficacy of ILT live vaccines in very young chickens [160].

In addition to the virus vector vaccine commonly used in chickens, DEV, which belongs to the family Herpesviridae, due to its large genome (approximately 158 kb), was used as a viral vector for the prevention of common viral or bacterial diseases in ducks. More importantly, DEV-attenuated live vaccines can rapidly induce protective immunity within several hours, and their efficacy is not affected by maternal antibodies [161]. These advantages make it gradually become a hotspot of vector vaccine research, which has been frequently reported in recent years, including being used as viral vectors to express the P. multocida outer membrane protein H (OmpH) [162], F protein of NDV [163], the hemagglutinin genes of H5 and H7 influenza viruses [164], and P1 and 3C protein of duck hepatitis A virus 3 [165]. All these recombinants had effective immune protection against specific pathogens. DEV vector is expected to be a powerful weapon to prevent common viral and bacterial diseases in ducks.

**Table 3:** Information on the recombinant viral vectors used as immunogens in chickens.

Vector	Foreign Gene Insertion Site	Pathogen	Antigen/s	Inoculation Route	Efficacy	Ref.
FPV	-	ILTV	gB	SC	Provided 100% and 70% protection for mortality and morbidity.	[83]
FPV	-	H7N3	HA	SC	rFPV-H7/3002 can provide 100% protection for mortality and morbidity. rFPV-H7/2155 cannot provide effective protection.	[166]
FPV	-	H5N1	HA and NA	WW, IM, or SC	Provided 100% protection for mortality via WW, IM, and SC.	[167]
FPV	Between the ORF161 and ORF162	NDV	F	WW	Provided 100% protection against FPV for morbidity. Provided 66.7% protection against NDV for mortality and morbidity.	[64]
FPV	-	H9N2	HA	WW	No virus shedding was detected.	[168]
FPV	-	IBV	S1	WW	Provided 100% protection for mortality.	[169]
FPV	-	IBDV	VP2	WW	Provided 100% protection for bursa of Fabricius Lesions.	[63]
FAdV-4	C terminus of fiber-2	H9N2	HA	-	Viral shedding was reduced.	[106]
FAdV-4	fiber-2	FAdV-8a	fiber	IM	Provided 100% protection for mortality.	[97]
CELO (FAdV-1)	Between two XbaI restriction sites	IBDV	VP2	SC or ID	Provided 100% protection for mortality and morbidity.	[113]
FAdV-8	Between two SpeI restriction sites or between SnaBI and XbaI restriction sites	IBV	S1	Oral	rFAV-S1 DA3 can provide 92.3% protection strain Vic S for replicating challenge virus in the trachea. rFAV-S1 CA6-20 can provide 100% protection against strain N1/62 for replicating challenge virus in the trachea.	[98]
FAdV-10	-	IBDV	VP2	IP, SC, IM	Provided 100% protection.	[100]
NDV	Between the P and M genes	IBDV	VP2	ON	Provided 100% protection for mortality and clinical signs.	[126]
NDV	Between the P and M genes	HPAIV H5N6	HA	ON	Provided 100% protection for mortality.	[141]
NDV	Replace the original NDV HN gene	NDV	HN	ON	Provided 100% protection for mortality and clinical signs.	[150]
NDV	Between the P and M genes	IBV	S1	ON	Provided 100% protection against NDV for mortality and clinical signs; Provided 90% protection against IBV for mortality.	[144]
NDV	Between the P and M genes	FAdV-4	fiber-2	IM	Provided 100% protection against NDV and FAdV-4 for mortality and clinical signs.	[96]
NDV	Between the P and M genes	IBDV	VP2	In ovo	Provided 100% protection against NDV for mortality. Provided 83.3% and 100% protection against IBDV for mortality.	[125]
NDV	Between the P and M genes	MDV	gB, gC, gE or gI	ON	The recombinant expressing the MDV gB protein provided about 90% and 70% protection against MD-induced tumor formation, respectively. Provided 100% protection against NDV for mortality and clinical signs.	[127]
NDV	Between the P and M genes	HPAI H7N9	HA	IN	Provided 100% protection against NDV for mortality. Provided 80% protection against HPAI for mortality and clinical signs.	[143]
NDV	Between the P and M genes	ILTV	gB, gC, or gD	ON	Provided 100% protection against NDV for mortality and clinical signs. rNDV expressing ILTV gD provided 100% protection against ILTV for mortality.	[148]
HVT	-	HPAI H5N1	Recombinant H5	SC or IM	Provided 100% protection for mortality (Except for MDA chickens).	[5]
HVT	-	IBDV	VP2	In ovo	Provided 100% protection for mortality and bursa of Fabricius Lesions.	[170]
HVT	UL45-46	Chlamydia psittaci	N-terminal fragment of PmpD protein	SC	Provided 100% protection against MDV for mortality, tumor formation, organ lesions, and clinical signs. Provided effective protection against Chlamydia psittaci.	[171]
HVT	-	HPAI H5	HA	SC	Provided 100% protection against homologous HPAI H5 for mortality and clinical signs.	[172]
HVT	US2	NDV	F	SC	Provided 70% protection against NDV for mortality.	[19]
HVT	Between HVT065 and HVT066 gene	HPAI H7N9	HA	SC	Provided 90% protection for mortality and clinical signs.	[173]
HVT	-	NDV IBDV	F and VP2	SC	Provided 100% protection against NDV for mortality and clinical signs.	[174]
HVT	Between UL45 and UL46 gene	NDV	F and HN	SC	Provided 100% protection against genotype IV NDV for mortality and clinical signs in SPF chicken. Provided 95% and 90% protection against genotype IV NDV for mortality and clinical signs in broiler chickens, respectively. Provided 100% protection against genotype VIIb NDV for mortality and clinical signs in broiler chickens, respectively.	[175]
HVT	Between UL45 and UL46 gene	H9N2	HA	SC	Provided 100% protection against H9N2 for clinical signs in SPF chicken. No virus shedding was detected.	[34]
HVT	US2	NDV ILT	F, gD and gI	in ovo or SC	Provided over 95% protection against velogenic NDV strain Texas GB for mortality and clinical signs. Provided over 85% protection against USDA strain ILTV 96-3 for mortality and clinical signs. Provided over 80% protection against MDV GA5 strain for clinical signs.	[16]
HVT	Between UL55 and UL56 gene	IBDV	VP2	SC	Provided 100% and 70% protection for mortality and clinical signs, respectively.	[31]
HVT	UL45–UL46	NDV	F	SC	Provided 100% protection for mortality and clinical signs.	[26]
MDV-1	UL41, US2, US10	IBDV	VP2	SC	Only r814US2VP2 provided 100% protection against vvIBDV for mortality, clinical signs, and bursal lesions. All rMDV provided 100% protection against vvMDV for mortality and clinical signs.	[22]
MDV-1	US2	ALV-J	*env* gene or *gag*-IRES-*env*	SC	Provided effective protection for viremia and development of the bursa of Fabricius in SPF chickens.	[36]

SC: Subcutaneous; WW: Wing-web stab; IM: Intramuscular; ID: Intradermal; IN: Intranasal; IP: Intraperitoneal; ON: Oculonasal.

## 3. Conclusions

Since the start of the 21st century, numerous recombinant live viral vector-based vaccine strains have been created and evaluated for their immunological effects against various poultry diseases. Compared with conventional vaccines, it has a significant advantage of long immune protection (Table 4). Although recombinant viral vector vaccines are gradually emerging and generally effective, the performance of some of the recombinant vector vaccines is affected by the presence of high levels of MDA in neonatal chickens. This factor has seriously affected the commercialization of recombinant vector vaccines. In contrast, HVT exhibits distinct advantages in evading maternal antibody interference and ensuring safety, which makes it stand out among other viral vectors. The widespread adoption of recombinant HVT vaccines in existing immunization programs merits consideration for implementation. For instance, administering HVT-based vaccines to 18-day-old embryos or 1-day-old chickens presents a promising strategy for safeguarding against ND, MD, and AI, which pose greater risks to young chickens. Furthermore, for viral infections that threaten older chickens, such as ILT or FAd, utilizing FPV-based vaccines may represent a better choice. Therefore, multivalent insert viral vector vaccine should be the focus of the next research phase. The wide application of recombinant live vector vaccines will greatly simplify the immunization process, save human and material resources, and improve animal welfare through fewer vaccine applications.

To further improve the immunoprotective effect, combining conventional and vector vaccines may address this problem. For example, compared with the administration of rHVT expressing the F antigen of NDV and the gD and gI antigens of ILTV alone (providing 88% protection), combining the recombinant vector with CVI988 improved the protection efficacy against vv MDV RB1B strain (providing 98% protection) [16]. In addition, according to the experimental results of Palya et al., administering rHVT-ND at one day of age, followed by immunization with a commercial live vaccine at six weeks of age and an inactivated vaccine at 15 weeks of age, significantly increased antibody levels against NDV and further reduced virus shedding [176].

Due to the extensive genomes of most viral vectors, constructing the corresponding reverse genetic systems poses a significant challenge, impeding the research and development of recombinant vaccines. In this context, utilizing a mini genome, a kind of engineered virus genome that does not contain non-essential genes for replication or virulence (preserving the antigenicity), presents a promising application. By significantly reducing the length of the viral genome, this approach facilitates the expedited development of a reverse genetic system to efficiently generate multiple recombinant vector vaccines. This strategy holds potential applicability to viruses such as HVT, MDV, ILTV, and FPV.

Over the past two decades, significant advancements have been made in utilizing recombinant viral vector vaccines within the poultry industry. Future endeavors should prioritize the incorporation of more recombinant live vector vaccines into immunization schedules and the strategic integration of various vaccine types and biological adjuvants with recombinant viral vector vaccines to confer robust immunity at a reasonable cost and prevent virus shedding. The forthcoming decade is poised to be an interesting period of progression for the research community dedicated to recombinant viral vector vaccines.

## Figures and Tables

**Table 1:** Characteristic of conventional vaccines and vector vaccines.

	Inactivated Vaccines	Attenuated Vaccines	Vector Vaccines
Safety	The best security.	Some have potential risk of virulence reversion.	Some have potential risk of virulence reversion.
Effect of the maternal antibody	Depending on the level of antibodies.	Depending on the level of antibodies.	Some can effectively avoid the interference of maternal antibodies, such as HVT-based vaccine.
Duration of immunity	Can only induce a short period of immune protection and requires multiple immunizations.	Can induce a long period of immune protection.	Can induce a long period of immune protection.
Immune response	The onset of protection was short but mainly induced humoral immunity. Some produced only a local response.	The onset of protection was long, but both humoral and cellular immunity could be induced.	The onset of protection was long, but both humoral and cellular immunity could be induced.
Cost	Less expensive	More expensive	Variable

**Table 2:** Advantages and disadvantages of each viral vector.

	Advantages	Disadvantages
MDV and HVT	Large genome containing several nonessential regions; Induce persistent infection for lifelong immunity; Avoiding interference from maternal antibodies; HVT-based vaccines have well-established safety profile.	The construction of recombinant viruses is time-consuming; HVT-based vaccines were less effective in preventing very virulent and very virulent plus MDV strains.
FPV	Genetically stable with low nucleotide mutation rates; Large genome containing several nonessential regions; Risk of recombination with the genes of its host organism; Hardly transmitting to other animals or disseminating into the surrounding environment; Lyophilized for storage.	Pre-existing immunity could inhibit the live vaccine from initiating an infection; Vaccine and field strains of FPV can be integrated with remnants of the REV.
FAdV	Convenient inoculation (can be administered orally); Lyophilized for storage.	The replication of FAdV serotypes was reduced in vivo due to the left-end deletion in their genomes.
NDV	Convenient and efficient reverse genetics system; Inducing both mucosal and systemic immune responses; Recombination events involving the NDV genome are exceedingly rare; The recombinant virus can acquire thermostability; Lyophilized for storage.	Maternal antibodies can affect the immune effect; Multivalent insert live vaccines are difficult to develop.

**Table 4:** Information of commercial conventional and vector vaccines against common poultry viral diseases.

	Conventional Vaccines	Vector Vaccines
NDV	AVINEW^®^ (Merial, Live Newcastle disease vaccine, VG/GA strain) can achieve 100% protection through aerosol. Protection until the age of 6 weeks.	Vectormune^®^ ND can provide protection against clinical signs, mortality, and drop in egg for layers until at least 72 weeks of age, following a single injection at day of hatch.
IBV	Poulvac^®^ IBMM (Zoetis, live infectious bronchitis vaccine, Massachusetts strain) can provide protection against Massachusetts serotype of IB for broilers and growing chicks. The duration of immunity may be 3–4 months.	There is no commercially available vaccine.
AIV	Inactivated bird flu vaccine (Yebio Bioengineering, H9 subtype) can prevent avian influenza caused by H9 subtype avian influenza virus. The duration of immunity is 60 days.	Newflend^®^ ND H9 can reduce mortality in the most susceptible period, clinical signs, lesions, and virus shedding caused by H9 subtype of low pathogenic avian influenza virus (LPAIV-H9). The duration of immunity is 9 weeks.
IBDV	Poulvac^®^ Bursa Plus (Zoetis, Live Infectious Bursal Disease Virus, strain V877) can reduce mortality and bursal lesions of Gumboro disease. The duration of immunity is 32 days.	VAXXITEK^®^ HVT + IBD can provide 100% protection against IBDV AL2 challenge strain at 21 days and up to 10 weeks duration of immunity following a single injection at day-old or in ovo. Immune protection against classic, variant, and vvIBD from 14 days of age.
MDV	Prevexxion^®^ RN can provide at least 80% protection against very virulent Marek’s disease. A single vaccination is sufficient to provide protection for the entire risk period.	Not applicable *
FPV	Yelive^®^ POX can prevent fowl pox with 5-month immune period for adult chickens and 2-month immune period for newly hatched chicks.	Not applicable *
ILTV	Rinbio^®^ ILT can provide protection against Infectious Laryngotracheitis for healthy chickens. The immune period is 6 months.	Innovax^®^-ILT can provide protection against Marek’s Disease and Infectious Laryngotracheitis. The immune period is 60 weeks.

* MDV, FPV is usually used as a vector for recombinant vaccines. Therefore, no information on commercial recombinant vaccines with one of its genes inserted into other vectors as a foreign gene has been found. More details on the commercially available vaccine: https://www.poultrymed.com/ or https://www.aphis.usda.gov/, accessed on 21 May 2024.
